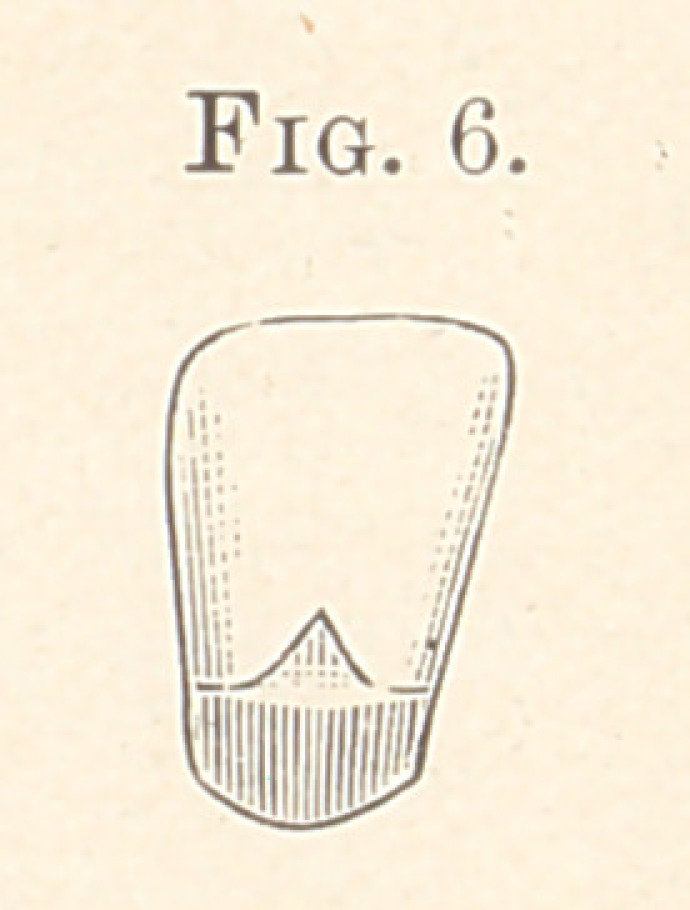# Crown- and Bridge-Work

**Published:** 1893-07

**Authors:** C. M. Richmond

**Affiliations:** New York


### International Dental Journal.

Vol. XIV. July, 1893. No. 7.

### Original Communications.[1]

### CROWN- AND BRIDGE-WORK.

BY DR. C. M. RICHMOND, NEW YORK.

(Continued from page 164.)

For using hot air for dental purposes, such as treatment of sensitive teeth, drying roots and root-canals previous to filling or crown-setting, various devices have been put forward having more or less to commend them, but the *ideal* device is yet to come. I have perfected one which has given more satisfaction than anything heretofore shown, and after a practical test of the one illustrated, I found that Dr. S. G. Perry, of this city, had made the same device in all its details, except the use of carbon, and with the same end in view. I found in my previous hot-air device (which was a hot platinum point through which the air passed) that though the first blast of air was of the required temperature, it at once cooled, while the platinum point was still hot. A blast of air would form a centre draft, and the hot air not being expelled, the cold air would make its way in its rapid passage through the hot air. This led to making a device which would cause the hot as well as the cold air to turn corners and thus keep perfectly commingled. The result is *perfect*, as a very hot blast of air and one of three or four minutes' duration is produced; so that one heating of the instrument is all that is required for an operation.

Referring to the cuts, Fig. 1 is an exterior view of the device in

[1] The editor and publishers are not responsible for the views of authors of papers published in this department, nor for any claim to novelty, or otherwise, that may be made by them. No papers will be received for this department that have appeared in any other journal published in the country.

full working size as I now use it, Fig. 2 is a cross-section near one side, and Fig. 3 represents the interior of the shell or case as it would appear if opened or made of metal; but I use for the cylinder a piece of hard round carbon having holes furrowed through it, as shown in Figs. 2 and 3; each end of the said carbon cylinder is capped with metal caps, as shown, and lined inside with disks of asbestos, which are cemented upon the ends of the carbon to hold them in position. The inlet and outlet tubes are made of metal and extend through the caps, as seen in Fig. 2, and are fastened by soldering or brazing. The nozzle may be made of platinum, if desired, and curved, as shown in Figs. 1 and 3. The inlet tube for the air is provided with a screw-thread to connect with the bellows or hand-blast, as most convenient. To give the desired heat for the air, I place the carbon cylinder, as shown in Fig. 1, in the flame of a Bunsen burner, and then it is or may be raised to a *red heat*, and as the air is forced through the curved passages, as shown in Fig. 3, it is thoroughly intermingled and perfectly completes the operation.

In Fig. 4 I have illustrated a piece of bridge-work for replacing the bicuspids and retaining the pulp alive in the cuspid as well as the molar tooth. The cuspid had been badly worn as well as all of the six front teeth. They were restored by contouring. In operating on the cuspid I cut with a small drill two holes on either side of the pulp-chamber to the depth of one-eighth of an inch. I then burnish a piece of pure gold, No. 30, over the end of the tooth, and the place where the two holes are will be marked in the pure gold. I then punch the holes where indicated and place through them a couple of tapering platinum pins, made for the purpose, and wax all together with a drop of hard wax. I now place it onto the tooth and crowd it down to position, forcing each pin as far as it will go into the holes and fitting the gold as near as possible. The piece is now invested, and the two pins are caught with a small piece of solder, just enough to hold them in place so as not to stiffen the piece of pure gold, which now has to be perfectly fitted to the end of the tooth while the pins are in position. I now put the piece in place, and with an automatic mallet and a foot-plugger (Butler's large preferred) the edges of the pure gold are set to fit the tooth-end perfectly. I now invest again and build up with solder to the required point. The gold molar having been fitted, the two porcelain teeth ground and waxed into position, and the gold tip placed onto the tooth, an impression of the case is taken with investment material (marble dust and plaster, equal parts, with a little salt added), and after it has hardened I remove it and place the parts in the

Fig. 1.

Fig. 2.

Fig. 3.

Fig. 4.

Fig. 5.

Fig. 6.

impression and cover with more of the investment. The whole case is now covered, and with a knife I cut into and expose the places where I wish to solder. After the case is finished I dry all the parts and the teeth and fill the two small holes in the cuspid; also fill the gold crown with sufficient cement to answer the purpose, placing the piece in position and forcing firmly home. After the case is cemented in position this gold tip is as perfect in appearance as if built on with gold foil and a mallet, and is much stronger, and will support one end of the small bridge perfectly, as shown in Fig. 4. In Fig. 5 I have illustrated a new porcelain crown for plate- and crown-work. This crown has a post or pin baked into it, and at the base of the crown are two diverging wings of platinum coming to the surface, as will be seen by referring to the cut. This tooth can be ground onto a plate or crown, and at once waxed into position and invested and soldered, without having to back with gold. A knife-edge of gold runs up into the split pieces of platinum and is attached to the crown or plate in the form of an oblong pyramidal shape, and instead of backing a tooth with a plate of gold, which robs it of its translucency, the gold runs direct into the tooth and supports it in the proper direction, thus saving a great expense and also much time and labor in backing and finishing.

Fig. 6 shows one of the new teeth attached to a band-crown, showing how small a piece of gold has to be finished after soldering. This tooth marks the first practical advance in porcelain teeth for crown- and plate-work for years, and is the most practical and strongest tooth yet made. It will be used at Chicago in giving clinics at the World's Columbian Dental Congress.

(To be continued.)

## Figures and Tables

**Fig. 1. f1:**
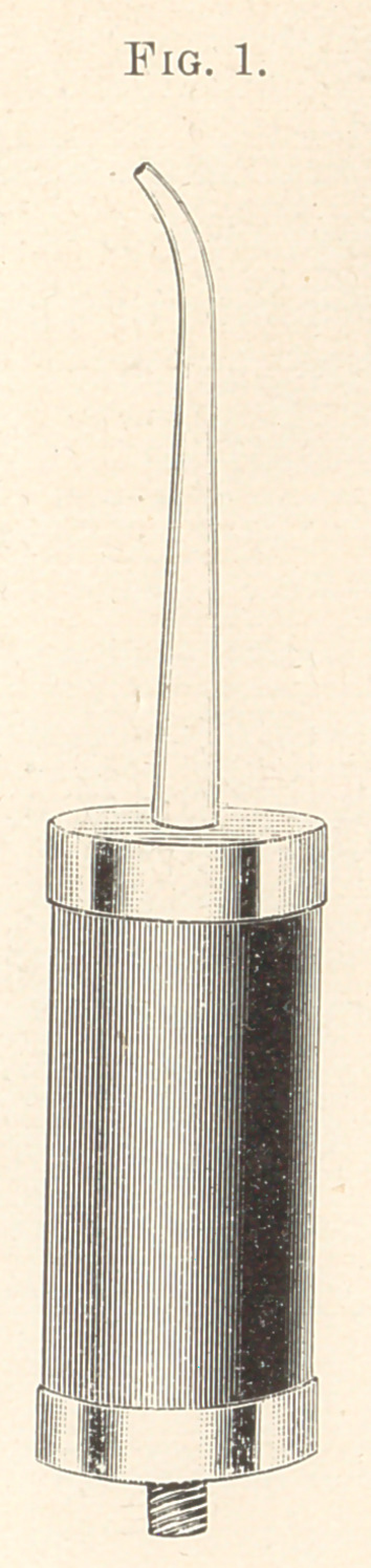


**Fig. 2. f2:**
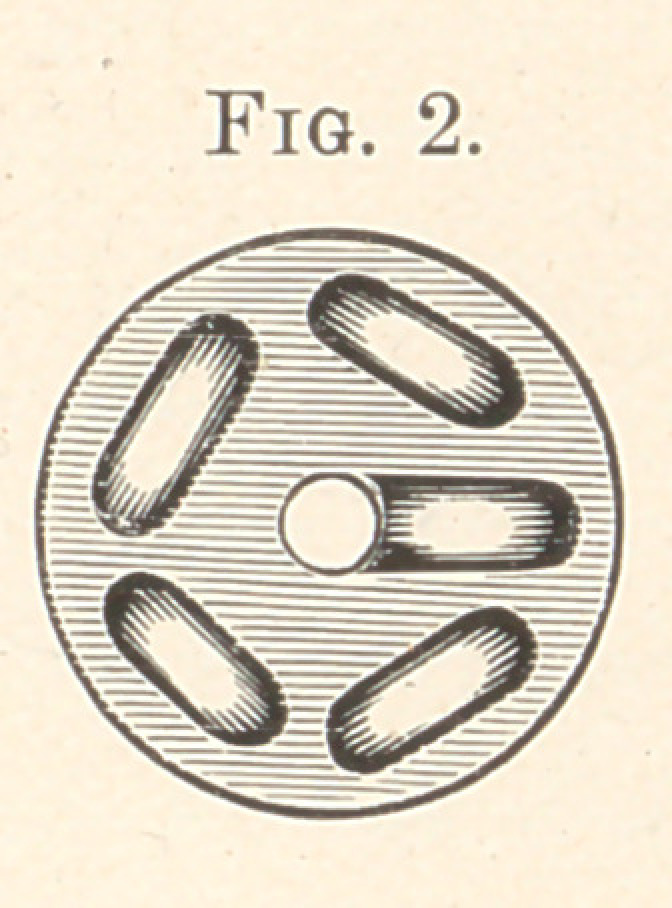


**Fig. 3. f3:**
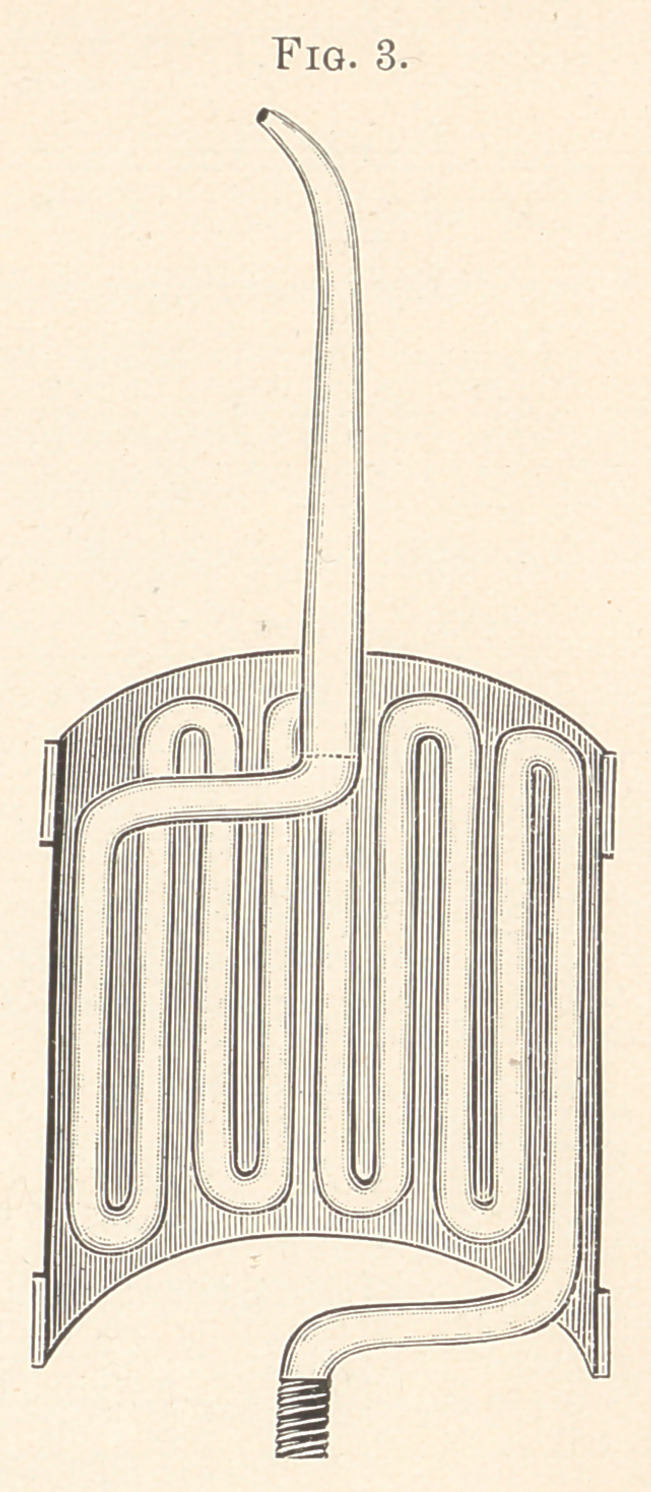


**Fig. 4. f4:**
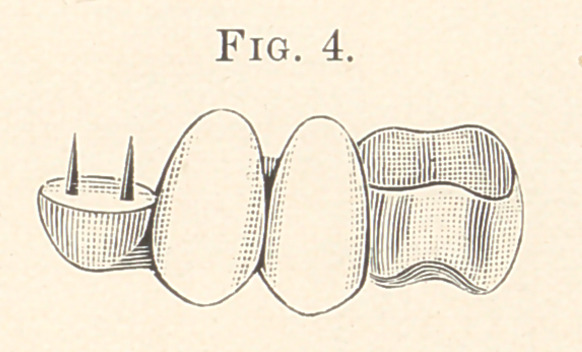


**Fig. 5. f5:**
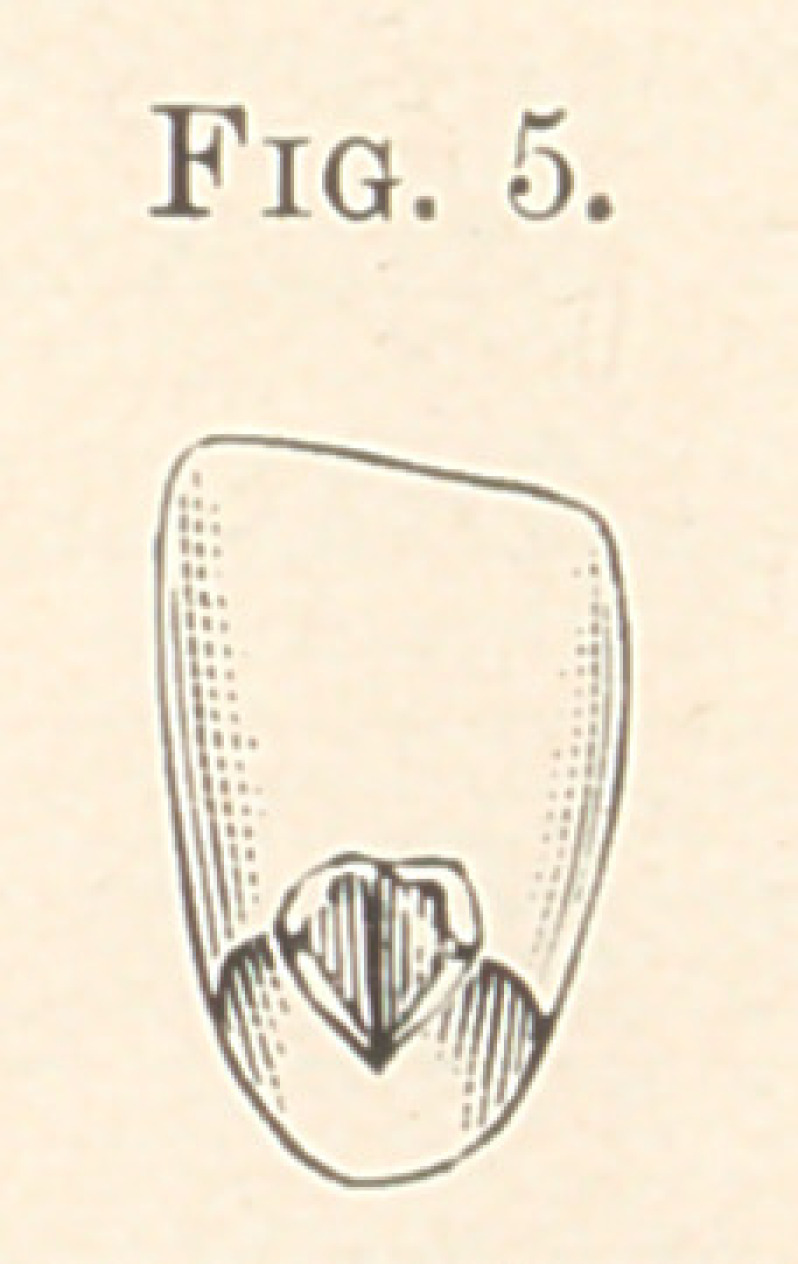


**Fig. 6. f6:**